# An Exploration of the Use and Impact of Preventive Measures on Skin Cancer

**DOI:** 10.3390/healthcare10040743

**Published:** 2022-04-15

**Authors:** Man Hung, Isaac Rex Beazer, Sharon Su, Jerry Bounsanga, Eric S. Hon, Martin S. Lipsky

**Affiliations:** 1 College of Dental Medicine, Roseman University of Health Sciences, South Jordan, UT 84095, USA; ssu@student.roseman.edu (S.S.); mlipsky@pdx.edu (M.S.L.); 2 School of Medicine, University of Utah, Salt Lake City, UT 84132, USA; 3 Huntsman Cancer Institute, Salt Lake City, UT 84112, USA; 4 College of Pharmacy, Roseman University of Health Sciences, South Jordan, UT 84095, USA; ibeazer@student.roseman.edu; 5 Merit Medical Systems, Inc., South Jordan, UT 84095, USA; jerry.bounsanga@utah.edu; 6 Department of Economics, University of Chicago, Chicago, IL 60637, USA; ericstephenhon@uchicago.edu

**Keywords:** skin cancer, sunscreen, NHANES, prevention, gender disparity

## Abstract

Background: Skin cancer is the most common form of cancer, and both clinical and epidemiological data link cumulative solar dosages and the number of sunburns to skin cancer. Each year, more than 5.4 million new cases of skin cancer are diagnosed, incurring a significant health and financial burden. Recommended preventive measures for skin cancer include the use of sunscreen, sun avoidance, and protective clothing. This study used a national database to examine the association of preventive measures with the prevalence of skin cancer, specifically analyzing the preventive measures of sunscreen use, staying in the shade, and wearing long-sleeved shirts. The second aim was to determine which characteristics, if any, correlated with using prevention measures. Methods: This study analyzed data from the National Health and Nutritional Examination Survey 2015–2016 cycle to examine the association of three preventive measures (using sunscreen, staying in the shade, and wearing long-sleeved shirts) with skin cancer. Logistic regression and chi-square tests were utilized to examine the relationship between skin cancer and these prevention methods. Results: Sunscreen use (OR = 3.752; *p* < 0.05) was statistically associated with a lower prevalence of skin cancer, while wearing long-sleeved shirts (OR = 6.911; *p* = 0.064) and staying in the shade (OR = 0.646; *p* = 0.481) did not emerge as factors significantly associated with a lower prevalence after controlling for gender, race/ethnicity, marital status, income, health insurance, and general health. Additionally, men and individuals of color were less likely to use sunscreen. Conclusion: Sunscreen use was associated with a lower prevalence of skin cancer, while wearing long-sleeved shirts and staying in the shade was not significantly linked to lower rates of skin cancer, suggesting that these measures may not be as effective as sunscreen for preventing skin cancer. Men and individuals of color were significantly less likely to use sunscreen. These findings can help guide future education efforts and research regarding skin cancer prevention and suggest the need to develop male-oriented programs to mitigate the gender disparity in employing sun-protection measures.

## 1. Introduction

Skin cancer is the most common cancer in the United States, with an average of 4.9 million adults treated annually [1]. Each year, more than 5.4 million new cases of skin cancer are diagnosed, outweighing the new cases of all other cancers combined [2]. Its widespread prevalence incurs significant health and financial burdens, and from 2007 to 2011, skin cancer treatment averaged an annual cost of $8.1 billion [1,3]. In addition, both melanoma and nonmelanoma skin cancer (NMSC) are associated with fatal outcomes [4].

Clinical and epidemiological data link cumulative solar dosages and the number of sunburns to skin damage and NMSC [5]. Preventive measures recommended to reduce skin cancers include physical barriers (e.g., hats, sunglasses, and clothing), sun avoidance measures (e.g., limiting sun exposure during peak hours and seeking shade), and using sunscreen [2,6]. The American Cancer Society and the Cancer Council Australia recommend all three measures as primary preventive activities [7,8,9]. Despite campaigns to promote these protective measures, studies indicate that many individuals fail to follow this advice, and the incidence of skin cancer continues to increase [10].

Most studies on sun protection focus on photodamage to skin deoxyribonucleic acid (DNA) as their primary outcome, and fewer studies evaluate the use effectiveness for reducing skin cancer as an outcome measure. Evidence demonstrates that wearing sun-protective clothing and avoiding sun exposure reduces exposure to ultraviolet (UV) radiation and the risk of sunburn [11]. Sunburn is often used as a biomarker of skin cancer risk, and research indicates that sunburn at any age increases skin cancer risk, suggesting that protective clothing and seeking shade reduce skin cancer risk [12]. However, data showing that these protective measures reduce skin cancer are more limited [13,14]. Explanations for this seeming paradox are that standard clothing materials may not always be effective in preventing the effects of UV radiation on the skin; that individuals may not consistently wear protective clothing; or that by seeking shade, individuals increase their total UV exposure by staying outdoors for longer periods [14].

Another strategy to prevent skin cancer is sunscreen, and for children, it remains the most frequently used sun protection measure [9]. While studies demonstrate that sunscreen reduces DNA photodamage, epidemiological studies raise doubts about its effectiveness in reducing skin cancer among the general population [15]. Although Green and colleagues found that regular sunscreen use reduced the incidence of melanoma [16] and cutaneous squamous cell carcinoma (SCC) [17,18], other studies found little protection [19], and some even observed an increase in skin lesions among sunscreen users [20]. Sunscreen also requires proper use, including application before sun exposure and frequent reapplication [21]. Use effectiveness may not match theoretical effectiveness since sunscreen use in real conditions may not be ideal [22]. Even when used properly, one study examining eight US Food and Drug Administration-approved sunscreen compounds found that when used individually, these compounds did not prevent squamous cell carcinoma [23], and a 2018 meta-analysis did not confirm the expected protective benefits of sunscreen against skin cancer in the general population [15]. A Cochrane database review also found insufficient evidence to conclude that sunscreen use reduced the risk of basal cell carcinoma (BCC) or cutaneous squamous SCC [21]. 

Sun avoidance—seeking shade and avoiding exposure during the peak hours of 10 AM to 3 PM—is also a commonly used sun protection method [24,25]. However, while this strategy reduces total UV exposure, its effectiveness in reducing skin cancer risk remains uncertain [25,26,27,28], and studies indicate that seeking shade alone does not decrease the risk of skin cancer [29]. Additionally, few adults regularly use more than one preventive measure [30], and it may be that those avoiding peak sun exposure may be less likely to combine this strategy with a physical barrier or sunscreen, thus limiting its effectiveness [21].

Using a national database, this study examined whether the use of three commonly recommended preventive measures—using sunscreen, staying in the shade, and wearing long-sleeved shirts—were associated with a lower risk of developing skin cancer. In addition, research suggests that characteristics such as gender, age, income, and ethnicity play a role in the utilization of sun protection [31,32,33,34]. Understanding the factors associated with prevention is important, and a second study aim was to determine which characteristics, if any, correlated with using sunscreen, staying in the shade, and wearing long-sleeved shirts. Identifying associations between using these measures and demographic characteristics can lead to more focused education and more effectively direct future intervention and prevention efforts.

## 2. Materials and Methods

This study analyzed data from the 2015–2016 National Health and Nutritional Examination Survey (NHANES) [35,36]. The NHANES is a survey conducted in two-year cycles throughout the 50 states and Washington D.C. that uses complex multistage probability sampling to represent the general, non-institutionalized United States population [35,36]. NHANES oversamples certain population subgroups to increase the reliability and precision of health status indicator estimates for these particular subgroups. The survey consists of two portions: an interview, including demographic, socioeconomic, dietary, and health-related questions, and a physical examination, including medical, dental, and physiological measurements and laboratory tests. Survey information includes data on the prevalence of multiple diseases as well as associated risk factors and exposures. The survey was administered to either participants or their proxies. Screeners interviewed participants who were 16 years or older and emancipated minors. For participants who were under the age of 16 years old and for those who could not answer the survey questions, an adult proxy answered on their behalf. Participation in the NHANES was by invitation only, and invited participants were required to live in one of fifteen designated counties in the United States during the data collection cycle. 

This study used the 2015–2016 survey as its data source, which consisted of 9971 individuals who were interviewed, 9544 of whom were also examined. After excluding 3979 participants under the age of 18 years old, the final study sample consisted of 5992 individuals aged 18 years and older.

### 2.1. Data Collection

The NHANES utilizes self- and proxy-reported personal interview data on various health conditions and medical histories for the medical conditions segment of the survey [35,36]. These questions were administered at home by the computer-assisted personal interview (CAPI) system using trained interviewers. The CAPI system contains consistency checks to reduce errors in data entry and utilizes online help screens to assist interviewers with terms in the questionnaire. Specifically, the dermatology section of the survey deals with questions regarding sun exposure and sun-protective behavior. 

### 2.2. Measures

Questions from the NHANES medical conditions segment consisted of “Ever told you had cancer or malignancy?” and “What kind of cancer was it?”, with options for skin cancer, such as melanoma, nonmelanoma, and unknown. From the dermatology section of the survey, questions regarding preventive measures were utilized for this study. These included “When you go outside on a very sunny day for more than one hour, how often do you stay in the shade?”, “When you go outside on a very sunny day for more than one hour, how often do you wear a long-sleeved shirt?”, and “When you go outside on a very sunny day for more than one hour, would you use sunscreen?” Options for answers consisted of “always”, “most of the time”, “sometimes”, “rarely”, “never”, “refused to answer”, or “don’t know” [35,36]. 

### 2.3. Analytic Approach

Demographics of participants diagnosed with skin cancer and those without skin cancer were reported using descriptive statistics, including means and standard deviations (SD). Logistic regressions were performed to explore the association of prevention methods with skin cancer. The three preventive methods included using sunscreen, staying under the shade, and wearing a long-sleeved shirt. Covariates included age, gender, race/ethnicity, marital status, income, health insurance, and general health. Chi-square tests were applied to examine the relationship between prevention methods and skin cancer. Additionally, sunscreen use by gender and race, staying in the shade by gender and race, and wearing a long-sleeved shirt by gender and race were examined using chi-square analyses. Univariate and multivariate analyses were also conducted to identify an independent effect of each of the preventive measures. Separate logistic regression analyses were conducted to evaluate the incremental value of staying in the shade and wearing a long-sleeved shirt to the use of sunscreen in the prevention of skin cancer. Odds ratios (ORs) were reported along with statistical significance, set at *p* < 0.05. All statistical analyses were performed using SPSS 25.0 (IBM SPSS Statistics for Windows, Armonk, NY: IBM Corp).

## 3. Results

The study sample totaled 5992 participants in the NHANES dataset and among the participants, 161 reported skin cancer. Table 1 displays the demographic characteristics of the sample. Of the 161 participants who reported having been diagnosed with skin cancer, 55.3% were males, 87.6% were Caucasian, and approximately one-third had a college degree. The average age was 69.5 years old (SD = 11.7; range = 20–80), over half were married, and 22% had a household income greater than $100 000. Of the 5165 without skin cancer, the average age was 47.7 years old (SD = 17.2; range = 20–80). The majority of those who did not have skin cancer were non-Hispanic with a high school education or above. Men experienced more skin cancer than women, with the difference in skin cancer between men and women approaching statistical significance (*p* = 0.07).

Among the study population with skin cancer, nonmelanoma skin cancer was the most prevalent type (Table 2) and was significantly more common than either melanoma (*p* < 0.05) or unknown skin cancer types (*p* < 0.05). However, the proportion of melanoma compared with unknown skin cancer types did not differ significantly (*p* = 0.52).

Of the 5992 participants, a subset completed questionnaires on skin cancer and preventive measures (wearing a long-sleeved shirt, staying in the shade, and using sunscreen). There were a total of 2224 participants who completed both the skin cancer questionnaire and the wearing a long-sleeved shirt question, 2769 who completed both the skin cancer questionnaire and the staying in the shade question, and 2915 who completed both the skin cancer questionnaire and the using sunscreen question. Among those who responded to the protective measure questions, males were significantly less likely than females to use sunscreen (*p* < 0.05), and Black participants were less likely than other racial groups to use sunscreen (*p* < 0.05). Regarding staying in the shade, females reported using this protective measure more often than males (*p* < 0.05), and Hispanic participants more often than other racial groups (*p* < 0.05). Males tended to wear long-sleeved shirts more frequently than females (*p* < 0.05), and White participants tended to wear long-sleeved shirts more often than other racial groups (*p* < 0.05).

Univariate analysis indicated that sunscreen usage (OR = 3.752; *p* < 0.05) was significantly linked to a lower prevalence of skin cancer, and staying in the shade (OR = 6.911; *p* =0.064) was marginally significant; however, wearing a long-sleeved shirt did not have a significant association with skin cancer (OR = 0.646; *p* = 0.481) (Table 3). After controlling for age, gender, race/ethnicity, marital status, income, health insurance, and general health, sunscreen usage was not correlated with a significantly lower incidence of skin cancer (adjusted OR = 0.956; *p* = 0.949) (Table 4), demonstrating that the effects of sunscreen use on skin cancer vary across demographics. Table 5 presents results from the stepwise logistic regression analysis showing that wearing a long-sleeved shirt (adjusted OR = 1.182; *p* = 0.813) did not add incremental value to sunscreen in the protection against skin cancer.

## 4. Discussion

Ideally, sun protection recommendations should be backed by strong scientific evidence. Evidence that the vast majority of skin cancers are caused by solar UV radiation exposure is accepted and provides the rationale for promoting sun protection [37] measures, such as sun avoidance, wearing long sleeved shirts, and using sunscreen. However, epidemiological studies raise doubts about the use effectiveness of these measures in reducing the incidence of skin cancer [15]. This study adds to the literature by providing comparative data on sun-protective behaviors and their association with skin cancer. A systematic review failed to identify studies that evaluated the benefits of sun-protective clothing or seeking shade when outdoors, and to our knowledge, this is one of the first studies to specifically examine these sun protection measures [21]. We found that sunscreen use was associated with a significantly lower risk of having all types of skin cancer, but this association varied across demographics. 

Our results indicate that while sunscreen use was associated with a significantly lower risk of having all types of skin cancer, wearing a long-sleeved shirt was not linked to a reduction in skin cancer. While the univariate results identified that staying in the shade was marginally associated with a reduced risk of skin cancer, there was an insufficient sample size to evaluate the multivariate association when controlling for confounding variables. One reason may be that these protective actions alter behavior, leading to spending more time in the sun and increasing overall exposure. Although sunscreen prevents sunburn, evidence that it prevents skin cancer is surprisingly sparse [15,38]. Some research suggests that sunscreen might paradoxically increase the risk of skin cancer by instilling a false sense of security that leads to an increased duration of intentional sun exposure [20,39]. While more recent research found that using sunscreen does not increase the risk of skin cancer, a systematic review failed to confirm the expected protective benefits of sunscreen against skin cancer in the general population [40]. Our findings add to the literature that does support the benefits of sunscreen as a preventive measure [41] by being one of the few studies to use a nationally representative US database to link a lower incidence of skin cancer to sunscreen use across all skin cancer types. Although they are observational and cross-sectional, the findings corroborate the seminal randomized control study demonstrating the beneficial effects of sunscreen use on skin cancer by linking sunscreen use to fewer skin cancers in the context of real-world use among a general population [16,17,42]. The cost of treating skin cancer is disproportionately increasing compared with other cancers, and in 2012 alone, providers received $77 billion in Medicare payments [43]; in addition to supporting a reduction in the clinical burden of disease, our findings support sunscreen use as an inexpensive means to help reduce rising healthcare costs. 

Although the reason why the findings reported here regarding sunscreen use may differ from earlier studies is uncertain, one possible explanation is the improvement in sunscreen formulations and UV blockage over the last two decades. In addition, public messaging and better product labeling might translate into improved application techniques with more consistent application, reapplication, and adequate amounts of sunscreen use. Further research is needed to confirm these findings regarding sunscreen. 

While they are promoted as preventive measures, unexpectedly, our findings did not identify seeking shade or wearing long-sleeved shirts as measures associated with a lower risk of skin cancer. One reason may be that shade only partially blocks UV radiation and depends on the shading structure and the amount of surface reflectance [44]. In addition, the effectiveness of clothing for blocking solar radiation depends on several factors, such as fabric density, thickness, material, and color [45]. The NHANES survey does not distinguish between clothing types, so it may be that individuals reporting wearing long sleeves might have worn clothing without good UV protection. Combining wearing long-sleeved shirts and sunscreen did not add incremental benefit to sunscreen use alone in preventing skin cancer. This finding suggests the importance of incorporating and perhaps prioritizing sunscreen in skin cancer prevention strategies. Similar to earlier research, this study found that men were about half as likely to use sunscreen either always or frequently when compared with women and adds to the literature about gender differences in attitudes and behavior regarding sun protection [46,47,48]. Skin cancer is more prevalent in men, and tailoring promotions to men to increase their use of sunscreen has the potential to reduce gender-related disparities in skin cancer incidence. 

Effective prevention interventions incorporate the dual mechanism of the decision to initiate protective behaviors and the inherent progressions to embed these behaviors as habits [49,50]. For skin protection, this includes recognizing the threat of skin cancer and the belief there are effective and easy behaviors to perform that reduce risk. For men, higher levels of masculinity are associated with lower levels of sun-protective behaviors and skin cancer prevention. One strategy might be to bundle sun protection interventions with other healthy lifestyle measures and, by clustering lifestyle factors, develop a comprehensive approach to health behaviors impacted by masculinity [33]. Interventions that may be more effective in men include incorporating humor [51], messaging that focuses on lowering the fear of skin cancer [52], providing free sunscreen, developing work-based sun protection programs, linking sunscreen characteristics to skin type, and clinicians unequivocally recommending that men use sunscreen. Male resistance to sunscreen use is also rooted in masculine cultural values and beliefs such as equating sunscreen with cosmetics and viewing sunscreen as a product more for women than men.

Of the racial groups studied, Black participants were less likely to wear sunscreen, a behavior that may be related to the perception that they are at low risk for skin cancer [53]. African Americans may also experience less access to sunscreen because of fewer drug stores and grocery stores selling sunscreen in African American neighborhoods and less sunscreen counseling by health care providers [54]. Although skin cancer is less common in people of color, when it does occur, it is often associated with increased morbidity and mortality [55,56]. Squamous cell carcinoma (SCC) is the most common skin malignancy among dark-skinned individuals [57], and since sunscreen may have a preventive effect on SCC [18], promoting sunscreen use among individuals of color has the potential to reduce the skin cancer outcome disparities seen in individuals of color. Older adults and individuals with less education also perceived themselves as being at a lower risk for skin cancer. To address these misconceptions, public health educational programs directed toward these demographics could also help to reduce skin cancer prevalence. 

Finally, the ultimate goal is to reduce the overall burden of skin cancer. The complexity of skin cancer prevention extends beyond just dermatologic issues and requires a multi-dimensional approach that incorporates an array of factors, such as the psychology of prevention, health perceptions about suntanned skin, the characteristics of target populations, attitudes toward applying chemical sunscreens, cost, social influencers, and indirect media messaging [58]. Future multi-disciplinary, collaborative research to explore these complex interactions can help reach the ultimate public health goal of reducing the burden of skin cancer.

### Limitations and Strengths

This study has both limitations and strengths. A key limitation is that since this is a cross-sectional study, the results demonstrate an association rather than establishing a causal relationship between a sun protection measure and skin cancer prevention. While randomized controlled trials (RCTs) provide a higher degree of evidence, they may take decades to detect a protective benefit from sunscreen use, and withholding sun protection from a control group raises ethical concerns. In the absence of RCTs, epidemiological observations such as those in this study offer useful information to guide recommendations. Another limitation is that the data were self-reported and subject to recall bias. However, self-reported sun protection data have been shown to be adequate, with correlations between self-reporting and observation ranging from 0.51 to 0.83 [59]. Data about the amount of sun exposure were also self-reported, but there is no reason to suspect any systematic bias among the sample groups. Each of the three racial groups used in this study (i.e., White, Hispanic, and Black) includes a wide range of skin pigmentation, and the data do not account for variation in pigmentation among these groups. By contrast, the study strengths include the use of a comprehensive database that draws a diverse sample of participants; in addition, this study is representative of the real-world use of protection measures among the general population. The collection of participant information also allowed adjustment for possible confounders. 

Since the relationship between sun exposure may differ by skin cancer type [60], the effectiveness of sun protection measures may differ as well. For example, it is possible that staying in the shade and wearing long-sleeved shirts afford protection to those with a family history of skin cancer, those with occupational exposure, others at high risk for skin cancer, or males. Analyses stratifying by skin cancer type were not performed because of the low sample size, especially for rarer types of skin cancer. However, public messaging promotes protective measures as being universally applicable to skin cancer protection, and our study assesses this message.

## 5. Conclusions

Sunscreen use was associated with a lower prevalence of skin cancer, while wearing long-sleeved shirts and staying in the shade or combining these protective measures with sunscreen use were not. This finding suggests the importance of incorporating and perhaps emphasizing sunscreen in skin cancer prevention strategies. Men, individuals of color, and those with lower incomes were significantly less likely to use sunscreen. These findings can help guide future education efforts for skin cancer prevention and highlight the importance of developing male-oriented programs to help mitigate gender disparities.

## Figures and Tables

**Table 1:** Demographic characteristics of participants.

Variable	All N (%)	Had Skin Cancer N (%)	No Skin Cancer N (%)	*p*-Value
Gender, n (%)				0.07
Male	2887 (48)	89 (55)	2476 (48)	
Female	3105 (52)	72 (45)	2689 (52)	
Race, n (%)				<0.001
Mexican American	1064 (18)	4 (2)	953 (18)	
Other Hispanic	798 (13)	8 (5)	708 (14)	
Non-Hispanic White	1914 (32)	141 (88)	1531 (30)	
Non-Hispanic Black	1265 (21)	3 (2)	1125 (22)	
Non-Hispanic Asian	726 (12)	0 (0)	667 (13)	
Other Race	225 (4)	5 (3)	181 (4)	
Educational level, n (%)				<0.001
Less than 9th grade	688 (12)	4 (3)	638 (12)	
9–11th grade	676 (12)	29 (20)	616 (12)	
High school graduate	1236 (22)	0 (0)	1129 (22)	
Some college or AA degree	1692 (30)	62 (42)	1501 (29)	
College graduate or above	1422 (25)	51 (35)	1278 (25)	
Marital status, n (%)				<0.001
Married	2885 (50)	96 (60)	2607 (50)	
Widowed	421 (7)	27 (17)	324 (6)	
Divorced	614 (11)	21 (13)	528 (10)	
Separated	192 (3)	2 (1)	165 (3)	
Never married	1048 (18)	9 (6)	1006 (19)	
Living with partner	555 (10)	6 (4)	533 (10)	
Annual household income, n (%)				0.32
$ 0 to 24,999	1544 (26)	35 (22)	1286 (25)	
$ 25,000 to 54,999	1648 (27)	53 (33)	1423 (28)	
$ 55,000 to 74,999	647 (11)	16 (10)	580 (11)	
$ 75,000 to 100,00	545 (9)	13 (8)	488 (9)	
$ 100,000 and over	955 (16)	34 (21)	826 (16)	
Missing	653 (11)	10 (6)	562 (11)	

**Table 2:** Distribution of skin cancer types in the sample (total N = 161).

Type of Skin Cancer	N	%
Melanoma	38	23.6
Non-Melanoma	75	46.6
Unknown	48	29.8

**Table 3:** Univariate analyses of the effect of preventive measures on skin cancer.

Variables	Has Skin Cancer (n)	No Skin Cancer (n)	Odds Ratio	95% CI	*p*-Value
Sunscreen use	17	791	3.752	1.784–7.892	0.001
Staying in shade	12	1403	6.911	0.897–53.246	0.064
Wearing long-sleeved shirt	3	497	0.646	0.192–2.175	0.481

**Table 4:** Multivariate logistic regression analyses of the effect of preventive measures on skin cancer.

Variables	Odds Ratio	95% CI	*p*-Value
Sunscreen use *	0.956	0.241–3.794	0.949
Staying in shade **	-	-	-
Wearing long-sleeved shirt *	1.182	0.298–4.693	0.813

Note: * Adjusted for skin reaction to sun after non-exposure, sunburns in past year, age in years at screening, gender, race/Hispanic origin w/NH Asian, marital status, educational level—adults 20+, annual household income, covered by health insurance, and general health condition. ** Unable to compute because of low sample size.

**Table 5:** Stepwise logistic regressions evaluating incremental value of “staying in shade” and “wearing long-sleeved shirt” to “sunscreen use” (dependent variable = skin cancer).

Variable	Odds Ratio (*p*-Value) 95% CI			
Sunscreen use	3.752 (0.001) [1.784–7.892]	1.174 (0.799) [0.342–4.037]	3.235 (0.008) [1.361–7.686]	0.956 (0.949) [0.241–3.794]
Staying in shade ^#^		-		-
Wearing long-sleeved shirt			0.610 (0.433) [0.178–2.096]	1.182 (0.813) [0.298–4.693]

Note: ^#^ Unable to compute due to low sample size.

## Data Availability

The data that support the findings are available from the National Center for Health Statistics.

## Abbreviations

BCC	basal cell carcinoma
CAPI	computer-assisted personal interview
DNA	deoxyribonucleic acid
NHANES	National Health and Nutritional Examination Survey
NMSC	nonmelanoma skin cancer
OR	odds ratio
RCT	randomized controlled trial
SCC	squamous cell carcinoma
SD	standard deviation
UV	ultraviolet
